# Measuring Farm Animal Emotions—Sensor-Based Approaches

**DOI:** 10.3390/s21020553

**Published:** 2021-01-14

**Authors:** Suresh Neethirajan, Inonge Reimert, Bas Kemp

**Affiliations:** Adaptation Physiology Group, Department of Animal Sciences, Wageningen University & Research, 6700 AH Wageningen, The Netherlands; inonge.reimert@wur.nl (I.R.); bas.kemp@wur.nl (B.K.)

**Keywords:** animal emotions, animal welfare, behavior, sensors, precision livestock farming, farm animals, animal-based measures

## Abstract

Understanding animal emotions is a key to unlocking methods for improving animal welfare. Currently there are no ‘benchmarks’ or any scientific assessments available for measuring and quantifying the emotional responses of farm animals. Using sensors to collect biometric data as a means of measuring animal emotions is a topic of growing interest in agricultural technology. Here we reviewed several aspects of the use of sensor-based approaches in monitoring animal emotions, beginning with an introduction on animal emotions. Then we reviewed some of the available technological systems for analyzing animal emotions. These systems include a variety of sensors, the algorithms used to process biometric data taken from these sensors, facial expression, and sound analysis. We conclude that a single emotional expression measurement based on either the facial feature of animals or the physiological functions cannot show accurately the farm animal’s emotional changes, and hence compound expression recognition measurement is required. We propose some novel ways to combine sensor technologies through sensor fusion into efficient systems for monitoring and measuring the animals’ compound expression of emotions. Finally, we explore future perspectives in the field, including challenges and opportunities.

## 1. Introduction

There are numerous benefits to studying animal emotions. Because we ourselves are animals, studying animal emotions can give us greater insight into our own psyche and how our emotions manifest not just physiologically but also behaviorally and cognitively. Additionally, studying the emotions of farm animals helps us to learn how to be better providers for our livestock, work animals, and pets. To be able to assess the welfare of farm animals thoroughly, better understanding of the affective experiences and emotions of the animals are absolutely needed. This understanding has tangible, practical benefits. For example, Weaver et al. [1] found that cows produced more and higher-quality milk when exposed to serotonin, a neurotransmitter linked to feelings of happiness and wellbeing. Happiness refers to a long-term positive state, while happy (happiness, happier) can also refer to the basic, discrete emotion “happy” [2]. In other words, happier animals have the potential to be more productive animals.

More broadly, learning about animal emotions has the long-term potential to give us better ecological insight than we have at present. Emotional cues from animals may give us an idea of the health of an ecosystem before major problems emerge. This may prove critical to conservation efforts in the wake of extreme climate change. In the agricultural sphere, our system has become highly industrialized over the last several decades. Smaller farms mostly disappeared and instead agriculture has grown massive in scale. These massive farms have heightened the challenges of identifying, monitoring, and caring for large groups of animals. This scale has also made keeping the animals satisfied mentally, and overall productivity of the animals more difficult.

While farmers may be open to utilizing technology to perform managerial and monitoring tasks, the possibilities of this field concerning emotions and mental states and its impact on animal welfare are not yet fully explored. There is tremendous potential in technological sensors for monitoring the emotional conditions of animals, allowing farmers to study behavioral changes, detect diseases [3,4], and easily make adjustments in care to promote the welfare of their animals and increase the yield on their products. To provide a high quality of life to animals, and to remove stress induced factors on the health and welfare of animals, monitoring and measuring of farm animal emotions becomes crucial.

The literature cited in this review article were collected using the Web of Science, Scopus, and Google scholar tools. To showcase the latest developments and recent findings of this research in this area and to narrow down the search, the authors restricted the search to papers published only in the past five years. Keywords used were, farm animal emotions; pig emotions; dairy cow emotions; sheep emotions; horse and chicken emotions; emotional contagion; animal empathy; animal emotions; sensors for emotions; sensor fusion and emotional contagion; measurement of emotions using sensors; sensors and farm animals; pain measurement in farm animals. Individual searches and Boolean were conducted as part of this study. Only farm animals were chosen from the pool of literature. The number of papers cited in this review is 129, with 20 that were published before 2015. The papers published before 2015 were included as the information on the sensing technologies for measuring emotions in farm animals were scant, and to signify the content on the need for sensors in the emotion measurement of farm animals.

This review identifies and describes the use of sensors technology to infer, analyze, and quantify the emotions of farm animals. We use the terms emotion, emotional state, emotional response, affective state, and emotional experience all interchangeably, and we mean the same in each case. This review first briefly covers the important aspects of emotion and how these are applicable to animals, then details different sensing technologies and analytical algorithms for monitoring animal emotions. In closing, it identifies future perspectives within this field, including challenges and unanswered questions.

## 2. Animal Emotions

One barrier to studying animal emotions is that the concept of emotion resists definition and quantification. There is no scientifically agreed-upon definition of what constitutes an emotion, and the term is often used interchangeably with others like disposition, mood, temperament, and mental state [5]. At its broadest, an emotion can be defined as a psychological phenomenon that helps in behavioral management and control, but this definition is too broad to be of immediate use [6]. More practically, the most commonly accepted definition is that emotions are biological states induced by neuropsychological stimulation brought on by physiology, behavior, and cognition [7]. Emotion is a state and not a trait. One useful framework for considering animal emotions is through the lens of affect or affective state. This is defined as the experiences and emotions that drive an organism to function. Affect drives animals towards reward and drives them away from punishment. In other words, affect connects the emotional inner life with the physical outer world [5].

**Figure 1:**
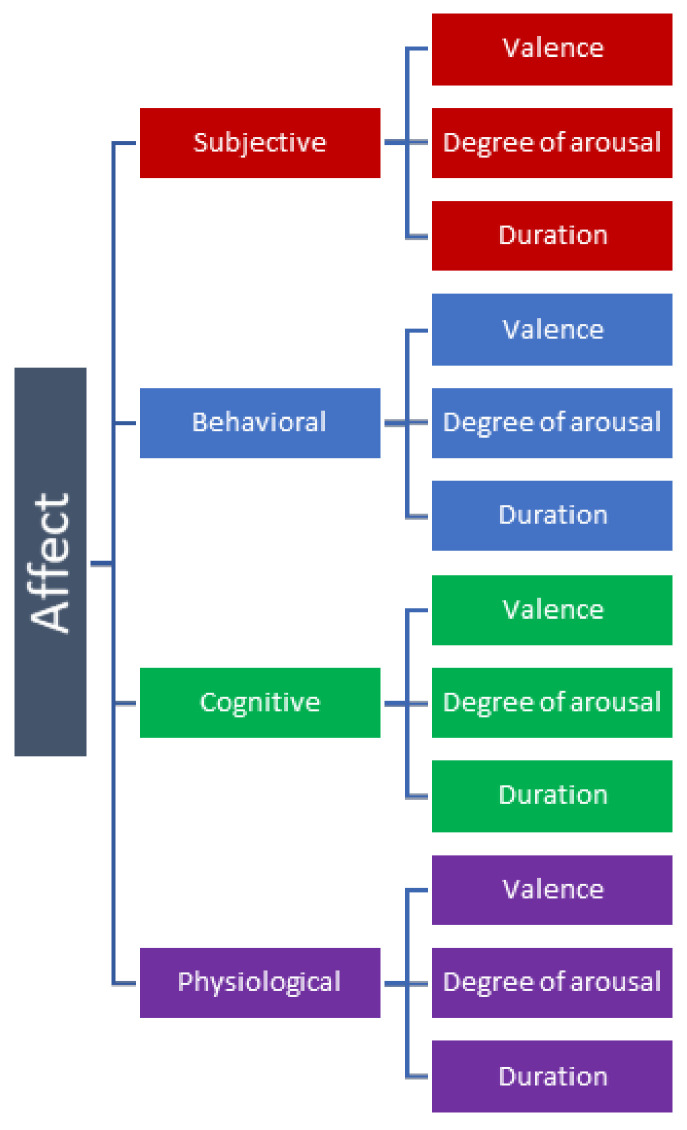
The framework of affective state.

Generally, emotions are considered to consist of four different components: Subjective, Behavioral, Cognitive, and Physiological [8]. Each component in turn has a valence, or direction—whether the experience is positive or negative. Furthermore, they may also vary in the degree of arousal and duration. This conceptual framework of emotion is illustrated in Figure 1. However, while we use this framework to discuss the matter here, the precise labels used to differentiate different components of emotions vary greatly among different research methodologies [5].

The Subjective component of emotion refers to the actual feeling experienced in real time by the given organism. Mammals (particularly primates) and birds experience something resembling emotions as we as humans understand them. Behavior in these animals is governed by automatic responses. The implication of this neurological hierarchy is that the subjective component of emotion can affect the behavior and emotional state of animals, but only those that have reached a certain level of cerebral organization.

The Behavioral component of emotion refers to how subjective experiences translate into tangible action. Some researchers argue that feelings cause behavioral changes, while others posit that it is the behaviors themselves that trigger feelings [9]. This component is complicated by the fact that behavioral responses may themselves feedback into the brain, causing further adjustment to the current emotional state. This is known as interoception, and it is not fully understood what effect, if any, this phenomenon has on animal emotions [10].

The Cognitive component of emotion refers to the way in which an organism thinks or makes decisions based on emotions. This component is also debated significantly in the field of animal science. Some researchers feel that cognitive processes and affective (emotional) processes are interdependent, while others believe the two systems are independent of each other [11]. It may be possible that affective processes may predate intellect, evolving from primitive subcortical structures [12].

Measurable aspects of the emotions including behavior, body language, sounds, facial expressions and physiological components are critical to the subject at hand in this review. The physiological component refers to the way that organisms experience bodily reactions in response to emotion. The role of the immune and neuroendocrine systems in emotions is well established in both humans and animals [13]. This paper deals with both the measurement of physiological and behavioral components (sound, body language including facial expression) of the farm animal emotions with a plea to combine these metrics.

### 2.1. Emotions and Animal Welfare

Animal welfare concepts call for all the activities involved in taking care of an animal physically and providing for its emotional wellbeing. Basic health and functioning; natural living; and affective states form the aspects/pillars of three circles model of animal welfare [14]. For example, a hungry animal may produce vocalizations or show a pronounced difference in posture, indicating a negative emotional state.

We know more about measuring animal welfare concerning the aspects of basic health and functioning and natural living but only a little about the measurement of affective state, particularly positive experiences such as pleasure and satisfaction. Maintaining positive affective states can lead to a greatly improved level of happiness and health for domestic and livestock animals [15].

Most farmers have significant emotional and financial incentive to take good care of their charges. Not only is this humane for the animal, but it also has distinct financial benefit since, as stated earlier, happier animals may be more productive in general. Emotion measurement is one concrete step farmers can take towards caring for their animals better [16].

An important consideration when designing a system for animal monitoring is to ensure that the system itself is not detrimental to animal welfare. Since no two individuals are alike, identifying individual farm animals is extremely important to this work. In the past, invasive methods such as branding, in-vivo sensors, and attaching transmitters with hooks or other invasive methods have been used. All of these may have negative impacts on the welfare of the individual in the form of infections, parasites, and emotional distress. They may also prove ineffective (e.g., transmitters can get lost). One of the great benefits of the emerging technology for animal monitoring is the potential for non-invasive identification. Today, software technology akin to facial recognition in humans has been developed for use in animal management and research. In the 1990s, visual and pattern recognition were combined with digital photography to create Visual Animal Biometrics (VAB) technology [17]. This technology gave us the ability to identify individual animals by their unique physical attributes, including even retinal patters, as no two animals have the exact same markings and colorations. This greatly enhances our ability to obtain accurate counts of populations, follow the animals’ movements, and provide for better welfare of both livestock and wild populations [18]. This recognition can be done remotely, without ever disturbing the animals, whether they are land-based, free-roaming, or aquatic [19]. So, while older methods of data collection sacrificed a small amount of good animal welfare for the benefit of the population at large, no such compromises need be made in future.

### 2.2. Common Emotional States in Animals and Their Presentation

Animals can experience and express a wide range of both positive and negative emotions [15]. Here, we briefly review the most common emotional states that researchers have studied in animals (Table 1).

#### 2.2.1. Pain

Pain is a dominant, aversive emotion in response to illness or physical injury in animals and it is distinct from human concepts of emotional pain (for example, grief) [20]. Pain in animals may elicit abnormal reactions, changes in motor skills and coordination, and unusual social behavior. Pain is commonly associated with production related diseases namely mastitis and lameness in dairy cattle and tail docking or castration in pigs.

#### 2.2.2. Fear and Aggression

Though animals may not have exact emotions that qualify as “anger,” they tend to be aggressive under certain conditions when they are pushed or provoked [21]. Traditionally, researchers have studied basic emotions such as aggression and fear in farm animals during the past two decades. Fear related studies focused on situations such as the animal facing a threatening situation, or presence of a predator or a novel object, etc. The aggression related experiments typically included conspecific interactions.

#### 2.2.3. Distress

Distress can include a variety of responses of the animal to a changing environment. In animals, it may present as changes in feeding habits, a compromised immune system, or elevated levels of the hormone cortisol [22]. Heat stress is a specific category of distress that occurs when the animal is unable to maintain the appropriate body temperature due to high ambient heat. Heat stress can affect fertility in animals [23]. Heat stress is also frequently accompanied by other health issues like dehydration. Frustration is another form of distress that occurs in animals when their access to a resource they need is cut off. This resource can be nutritional, like food or water, or it can include resources such as access to mates or mating habitats.

### 2.3. Physiological Indicators of Emotions in Animals

Using physiological cues or biomarkers to monitor animal emotions is just as important as monitoring visible behavior. Although, physiological cues are generally considered less informative on the valence facet of emotion [5], the benefit of using these types of cues is that they can be tracked chemically through biosensors, allowing for a more objective, quantitative analysis of the animal’s emotion, including inferences about the arousal facet.

In humans, for example, states of anxiety and or tension are related to elevated or augmented blood lactate levels [48]. Blood lactate concentrations in livestock indicate the severity of stressors and underlying disease conditions such as respiratory diseases or neonatal diarrhea, or displacement of abomasum [49]. In beef cattle, such states have been studied using cortisol measurements in the hair matrix [50,51]. Cortisol concentration in saliva has been used as a biomarker for changing stress levels in pigs [52]. Chewable silicone stick-based (popsicle or lollipop) sensing devices have the potential to measure salivary concentrations in pigs and cattle. Salivary oxytocin in pigs, cattle, and goats have been shown to influence positive human-animal interactions and can be an effective biomarker of positive emotions [53,54].

Measuring sample matrices and biochemical signatures in urine, nasal, and saliva secretions of farm animals indicating emotions is not well established. Serotonin (5-hydroxytryptamine, 5-HT) is a prototypical neuromodulator and significantly impacts animal cognition and behavior, and this neuromodulator is fundamentally involved in the adaptation of animals [55]. Because dopamine cannot be directly measured, researchers opted to measure catecholamines in livestock as a stress indicator. Increased level of catecholamines in beef cattle is considered as an objective measurement of pre-slaughter stress in cattle [56]. However, it should be noted that there are no commercially available on-the-spot measurement devices or sensors for any all these biomarkers.

### 2.4. Reading Animal Emotions

One of the greatest challenges of monitoring animal emotions is that many of the methods that might prove useful for humans, such as surveys or interviews, are useless for creatures that cannot read, write, or speak. In addition to language, humans also have a strong body language. The relative ease with which it is possible to identify human emotions has led to several technological systems for sensing human emotions [57].

Most animals, with the arguable exception of monkeys and apes, lack these mechanisms and must rely on other means to convey their emotions. Some animals use vocalizations such as growls, murmurs, barks, roosting calls, or purrs. Other animals use tails and body posture, like wagging a tail when happy or swishing a tail to convey anger. These types of signals can communicate, or even spread, the animal’s emotions within species or to humans [58,59].

However, such signals are not without fault. Even among humans, they are often misinterpreted, leading to embarrassing, and sometimes unfortunate results. A major complication in reading these signals is that the majority of animal behavior and physiology studies are not done on free range or wild animals. Rather, they are usually undertaken with domesticated or captive animals.

In general, emotional states fall into two main categories as described by Jaak Panksepp [60]; primary and secondary emotions, and each can be further categorized into positive and negative states. While primary emotions are generally easier to interpret, since they are based upon instinctual responses and thus may be similar across individuals of a species, secondary emotions are more nuanced. Interpreting farm animal emotions thus requires a solid knowledge of the species in question, as well as familiarity with the individual.

### 2.5. Relationship between Emotions, Facial Patterns, and Sounds

Two behavioral indicators of emotion relevant for sensor technology in farm animals are facial expressions and sounds. The ability to connect the face and sounds of an animal to an emotional state is critical for many practical applications, due to the fact that most livestock animals are mammals capable of changing their facial expression to a certain degree. An early study of the relationship between the expression of the face and emotions was published in 1964 [61]. However, it and many of the studies to follow were focused primarily on human emotions rather than animals.

Today, the scope of the research has expanded, and facial expressions are widely considered to be a great means of assessing the internal state of an animal. Pain expression is difficult in animals, and research is only now emerging on the use of facial changes in response to pain or stress [62]. Horses in particular have also been shown to have positive facial expressions [63]. One challenge of these types of studies is the difference in indicators of fear and stress within a species, overlapping of emotions, and false indicators based on other, unknown stimuli.

Farm animals also convey emotional states through sound. Sounds have been demonstrated to be indicators of emotions in several animals including horses [64], pigs [65], poultry and cattle [66]. Many animal vocalizations, particularly those indicating a negative emotion, are involuntary. This suggests that sounds may often indicate primary emotional responses as a first reaction.

## 3. Technologies for Measuring Animal Emotions

At present, direct measurement of emotion (as in the subjective component) is not possible, even for humans. Indirect measurements of emotion are time-sensitive and are difficult to take manually. However, modern technology is making observation and analysis of animal behavior and physiology faster and more effective. In this section, we discuss different technologies for monitoring farm animal emotions, including sensors, facial expression, sound analysis, and multimodal integrated technology approaches.

### 3.1. Sensors

Visual sensors (cameras) and biosensors constitute a significant part of the solution to automate the monitoring process of farm animals [67]. Sensors and biosensors in this context refer to devices that collect data about a specific physical, chemical, biological or biochemical parameter that can then be measured and analyzed [19].

Sensors can be affixed to a part of the barn, placed in a grazing field, or placed on or implanted within the farm animals themselves. They can be classified as wearables or non-wearable remote types and are invasive or non-invasive depending on the location. Noninvasive sensors are those located external to the animal, immobile, and nonattached. Alternatively, they can be attached to the animal’s body to collect information [68].

Invasive sensors are those that are implanted into the animal. While invasive sensors can provide more accurate, individualized data, they may induce stress that skews the data or harms the animal’s welfare, so these sensors must be used carefully or avoided. Biosensors can be invasive or wearable and non-invasive and detect the presence of specific biological compounds, such as a hormone or enzyme [67]. Each category of sensors has its benefits and drawbacks, and each can be used to attempt to quantify the emotional experience of the animal. While wearable sensors are frequently more accurate in terms of the parameter they measure, they also require large numbers of individual sensors to get a sufficient dataset to assess the emotional state of all individual animals. On the other hand, a small number of immobile sensors can be used for a large number of animals, as long as they are placed in locations where the animals will frequently be present.

There are several categories of sensors commercially available and are under development, and each measures a distinct parameter and has its own benefits and drawbacks (Table 2).

### 3.2. Global Positioning System

The global positioning system (GPS) is satellite-based standard sensing technology used for tracking farm animals’ location. Despite its longevity, initial cost of installation and implementation of this technology is quite high [69]. GPS sensors continuously monitor and maps the places the animals wander. These data can then be used to draw conclusions about the collective habits of animals in a group, or of individual animals. GPS is the primary tool for behavioral insights in grazing animals. GPS is also useful for monitoring wild animals, making it a frequent choice for measuring emotions in farm animals [70]. RFID and UWB are terms more often used in farm animals kept indoors than GPS.

However, GPS is not without limitations. Battery life, accuracy, and loss of data due to noise or external factors are all issues that may arise with a GPS tracking system. Despite these limitations, GPS is still widely used. Interestingly, Fogarty et al. [4] found that GPS was the most frequently used type of sensor to study sheep but was not used in studies on sheep welfare. This suggests that location data are at present not the primary parameter in the measurement of emotions of livestock.

### 3.3. Thermal Infrared Imaging Sensors

Thermal imaging captures images of animals using infrared light as opposed to the visible spectrum [46]. This results in an accurate indicator of temperature throughout the animal because infrared radiation is directly linked to heat. In order for this system to function, it must be able to continuously and precisely monitor body temperature. But once that is successful, it is a valuable tool. Additional benefits of infrared radiation include the fact that the sensors are no more invasive or destructive than a regular camera [70].

Thermal infrared imaging has been successfully used to detect pregnancy, measure heat stress, monitor foot lesions in cattle, and detect diseases like bovine respiratory disease complex and foot and mouth disease [71]. Interestingly, it is likely that thermal imaging may even be used to measure emotion. For example, changes in nasal temperature in cows have been associated with positive emotional states [48], eye temperature has been used to evaluate stress in meat goats [72], in combination with behavioral data temperature of the inner corner of the eyes that seems to be related to stress and negative emotions in sheep [73].

### 3.4. Electrocardiography

Electrocardiography (ECG) is a system that measures the electrical potential difference between two electrodes that are placed at the opposing ends of the cardiac flow, effectively measuring the electrical activity of the circulatory system. A third neutral electrode is set to remove the noise or the readings from other animal systems to give accurate results [74]. The recurring cardiac flow pattern is measured to monitor the functioning of the heart. Emotional reactivity, such as avoidance of other cows, can be reliably measured from the baseline values of the changing heart rate [75]. ECG systems greatly simplify the task of monitoring livestock and detecting problems with the heart and respiratory system. Based on the results of this monitoring, preventive measures or actions can be taken to handle the problem if needed. One major disadvantage of ECG monitoring is that it is generally not possible to continuously monitor animals with ECG. Often, the system is only employed when there is already probable cause to suspect a health issue. Currently, research is underway to design and develop wearable non-invasive ECG sensor systems for humans and these sensors will only need a few iterations before being able to adopt for farm animal applications.

### 3.5. Heart Rate Variability

Heart rate variability (HRV) is typically defined as variation in the beat-to-beat fluctuations of the cardiac cycle length under normal sinus rhythm [76]. Unlike ECG, there are portable systems available for storing heart rate variability data [70]. Two electrode rods are placed for optimal readings with a specific transmitter for horses and cattle. Different sized electrodes are available for smaller animals like calves and sheep [67]. There are also different systems for recording HRV in farm animals that require restriction in the movement to avoid motion artifacts in collecting data. These systems have been tested on poultry, pigs, and goats [77]. Differences between inter-beat intervals of heart rate along with vocalization sensing data have been shown to objectively assess emotional valence in pigs [78].

Heart rate variability has been extensively used in studies to research sympathovagal balance as it relates to stress, emotional states, and temperament of farm animals. For instance, postpartum fever in dairy cows is directly proportional to increased heart rate [79]; pigs’ stress response to heat episodes has been shown to be evaluated by heart rate variability [80]. Besides sympathovagal balance, the inter-beat interval (IBI) has been used in diagnosis of certain cardiac conditions as well as monitoring stress and anxiety in farm animals. The IBIs are coded to avoid data corruption from other readings in the area [80].

### 3.6. Electroencephalography

Electroencephalography (EEG) is a critical technique for pain research and nociception [81]. Much like ECG, EEG uses electrodes to monitor electrical activity within the body, but EEG targets the brain instead of the heart. Animals must be anesthetized before being subjected to EEG, but once the electrodes are in place EEG can provide an accurate reading of the brain activity irrespective of the movement of the subject. Currently, EEG has been particularly useful in measuring stress in animals [82] as well as responses to noxious stimulation [83]. Emotions in humans and non-human animals can be recognized through correlation from brain activity with the help of EEG signals [84,85] since EEG is also useful for emotion measurement, considering that it can be used on animals right up to the point of slaughter.

One weakness of EEG is the dissociation between mental states and EEG readings. This is to say that not every emotional state produces a distinct reading, so analysis requires objective knowledge of principles of EEG and its correlation to physiological functions and emotions of animals [86]. Figures of merit and additional validation and benchmarking need to be established through research to overcome the adoption of EEG as a wearable sensor for measuring the activity of farm animal brains.

### 3.7. Electromyogram

An electromyogram (EMG) measures the electrical activity of the muscles. It detects the electrical impulses produced by skeletal muscles. EMG has proven a useful technique to study muscle activity during pregnancy in sheep and humans [87]. It has also seen use in invasive and noninvasive evaluation of equine performance and muscle condition [88]. These data are especially important for labor animals like horses, because understanding the muscle activity of horses facilitates training.

This technique is used sparingly, as it records only superficial muscle activity and is subject to interference from many ambient factors such as temperature. Additional barriers to the extensive use of EMG are the difficulties in establishing solid benchmarks against which to compare experimental subjects [89]. In general, EMG is a useful research and diagnostic tool, but not yet applicable for day-to-day monitoring of muscle activity of animals on the farm and thereby it could be used in animal emotion research. The potential link of muscle activity such as tensions in muscles when the animals are in a fearful state has yet to be explored through sensors technology.

### 3.8. Respiratory Rate Analysis

Respiration pattern such as the velocity and depth indicate changes in emotions [90]. Respiratory rate (RR) analysis is a veritable tool in the arsenal of farmers; however, it is a time-consuming process that consists of monitoring flank movements to measure RR. Due to the sheer number of animals usually present on a farm, this is not a practical method for day-to-day monitoring. However, respiratory rate is a reliable measurement for medical diagnosis and research [57], for instance an increase in RR is indicative of high stress and potential illness in animals. Moreover, RR is also useful for animals with characteristic respiratory patterns, like dogs [91], and could be employed in pigs and dairy cattle as well.

An ideal RR sensor should be differential, pressure-based, transmit continuously, and sustainable. As it works today, RR is not constant and is influenced by factors like heat, high milk yield, and physical activity [92]. Using RR systems over the long term may prove to be useful in animal emotion research, but for this more research is needed. Thus, the abundance of interfering ambient factors and conditions make this, at present, an unreliable technique when used in isolation. That said, RR is an excellent complement to other sensor measurement systems.

### 3.9. Olfactory and Chemical Sensors

Olfactory and chemical sensors have tremendous potential in assessing animal emotion because animals use their sense of smell for a variety of essential processes: searching for food, sensing danger, and even determining when and with whom to mate [93]. Sense of smell is also well-connected with emotional and social responses in humans and animals [94]. Many farm animals have superior olfactory senses. Pigs, for example, are known to have an excellent sense of smell, but grazing animals like sheep or goats also have an exceptional sense of smell, which they use to avoid toxic plants and weeds [40,95]. Chemical and olfactory sensors monitor animals indirectly by detecting chemicals external to the animal. Chemical sensors may also be used to monitor chemicals present in bodily excretions like saliva. Olfactory sensors can also provide an early intervention for certain animal disease, like flystrike in sheep [96]. In this way, olfactory sensors could provide information on the overall state of the farm as opposed to consistently monitoring individual animals. Emotional states are also reflected in the odours of animals. For example: fearful pigs emits ‘alarming substances and volatile metabolites’ (allelochemics) that can be smelled by other animals [97]. Odour cues and olfactory awareness expressed by animals can be measured using sensing platforms to understand the correlation between the emotional states and the expression of various allelochemics. Adoption of sensors based analytical tools may be a game-changer in using odour as a biomarker for determining farm animal emotions through decoding the social volatilome.

### 3.10. Sound Analysis Sensing Platform

Sound analysis is a well-researched, sensor-based method for measurement of emotions [98]. Precision livestock farming with sound analysis is relatively easy to implement. Sound analysis sensing platform is comparatively more manageable to set up than other sensors, since the sensor itself consists of a simple audio recorder. The sensor is fixed to one location and records ambient sound. Therefore, this method can use a single sensor to monitor many animals [99].

The field of bioacoustics, or the extracting of valuable biological information from sounds makes this effort possible. Sound analysis has been successfully undertaken with pigs [100], poultry [66], and cattle [22]. The animals are first placed in situations that trigger certain vocal responses. Neuroscientists have shown the interconnectedness of neurons and the physiology and expression of emotions [101,102]. The neural and physiological responses expressed in the form of vocalizations in the farm animals are then measured. It is assumed that fearful or stressful situations may evoke negative emotions, which allows this benchmark measurement to be used to identify this emotion through comparison later. It is easiest to use a sound analysis system in animals without a large range of vocal sounds.

In pigs, stress such as throat, heat, and cold stress was found to be easily measured, as there is not much vocal modulation [100,103]. In addition, piglets seem to indicate through vocalizations when they are in pain or hungry [100]. Sensor-based vocalization data has to overcome the interference from ambience, and hence the filters for signal processing play a vital role in creating insights. Another example of where sound analysis is used is in the health management of broilers chickens. When suffering from respiratory diseases, the broilers tends to make an abnormal sound like coughing. Sound analysis was found to be efficient in identifying stress, diseases, and behavioral changes in these animals. Additionally, this is a technique that can also be implemented in a closed commercial building such as barns or pens, rather than an open space farm [104].

## 4. Emotional Facial Expression

Facial expression technology is already used frequently in human applications [105] and has the potential for use in animal emotion research. Although the facial features of farm animals are not fully established and associated with emotional states, this is a growing field of research. Each small movement in the eyes, ears, nose, cheeks, and jaws of a livestock animal may signify a varying emotion.

For instance, it has been observed that in sheep when isolated or in other unfavorable conditions, the aperture of the eyelids increases [106]. Similarly, when in fearful or stressful situations, there is the widening of the eyes and an increased visibility of the white sclera in horses [107], pigs [78], and cows [32]. Horses also display signs of pulling the upper lip when afraid [108]. In horses, yet another significant facial action is yawning. The frequency of yawning by a horse is related to both positive emotions and the performance in typical behaviors induced by frustration and repetitive habits [40]. In addition to changes in eye size, posture changes of the ears may be indicative for certain emotions in farm animals. For example, half closed eyes and ears hanging down in dairy cows could indicate that they are relaxed [33]; on the other hand, backward ears in pigs may indicate fear [44,45]. Besides identifying emotions in individual animals, facial expression analysis has the potential for larger-scale monitoring [109]. On farm pig face recognition tools have been recently developed by our research group [110,111] using non-invasive imaging sensors. The facial recognition platforms enable the possibility of identifying individual animals without the help of RFID tags.

### Facial Expression of Pain

Management of pain is critical to the improvement of animal welfare and using facial expression is currently considered a promising tool to assess pain in farm animals [25]. The current grimace scale scoring systems, in which several types of so-called facial action units are evaluated in establishing pain levels, combined the general function of the body, physiological responses, and behavior observation [112]. In addition, facial action coding systems (FACS) for various animals have been developed in the last few years, and they have provided an objective method to identify all possible facial expressions of an animal and make comparisons between species [112].

Although it seems that pain can be quite well assessed from animals’ faces, it should be mentioned that pain is subjective and depends on the individual animal and its tolerance to pain. Therefore, one significant challenge in using facial expression for pain measurement is the establishment of reliable benchmarks. There may also be interference from false positives, or a facial expression that was not caused by pain. It should be emphasized that pain of farm animals are still measured by subjective scoring, but can be improved by automated detection through sensing technologies.

## 5. Algorithms for Biometric Data Processing

Investigations of correlations between physiological parameters such as heart rate variability, skin conductance (electrodermal activity), and skin temperature changes (infrared data), and facial-emotional responses characterized using multisensor complex data would need significant computing power. Distinguishable expressive characteristics of the animals from the sensor data based on a multitude of physiological functions, and the dynamic changes of their emotional states over time requires the ability to systematically extract information from big data.

Data gathered from sensors are only part of the process of analyzing animal emotions. Another part of the process is about algorithms that can be used to analyze the data gathered. Choosing the best algorithm is a challenging task. In general, these algorithms rely on the modern capacity of computers for machine learning (ML). Machine learning is the capacity of computer algorithms to learn and adjust themselves as more and more data is loaded into the system. Machine learning systems have wide-ranging applications, including in agriculture [113]. The systems share the ability to take in all available information (facial expressions, temperature readings, vocalizations, etc.) and establish benchmarks, then compare these benchmarks to new information to sort out inconsistent results. The result is akin to a living, breathing machine. There are hundreds of machine learning algorithms available to analyze data from sensors. These can be split into three main categories and the key features of each system are summarized in Table 3. To be able to predict and often estimate the affective states of an individual through physiological functions and behavior, machine learning techniques are required. Due to the large volume of data, and in the feature selection and optimization of emotional parameters such as valence, duration, and activation, ML algorithms comes in handy [114].

### 5.1. Neural Networks

Neural network systems use many distinct machine-learning algorithms working together, forming a problem-solving system that mimics the human brain’s capacity for multidirectional thinking and pattern recognition. They can handle variable inputs with relative ease, which is important for consistent monitoring. Neural network systems also have the capacity to simulate the brain of the target species to optimize results without the need to consistently redesign outputs. Neural network systems have been successfully implemented in various ways in studies with sheep [115], pigs [116], and cattle [117].

### 5.2. Fuzzy Logic

Computer algorithms usually rely on only two value states—true (1) and false (0). By contrast, fuzzy logic is a system that introduces multiple values—states between true and false—that are similar to the human thinking process and closer to human intuition than facts [118]. By setting values for known states like a typical facial expression, fuzzy logic can be used as a reliable tool to compare continuous inputs to known baselines. Like all machine learning systems, fuzzy logic systems will flush out inconsistencies as more and more data are analyzed. Fuzzy logic has proven useful for detection and diagnosis of diseases and has been implemented in sound analysis systems in pigs [119].

### 5.3. Support Vector Machine Algorithms

This machine learning model is used primarily for classification problems. The algorithm works by plotting ‘n’ features on n-D space and determines a hyperplane to divide them. More simply, it takes the number of features in question (e.g., presence of specific characteristics in certain parts of the face) and plots them into a mathematical space of that many dimensions before dividing them into categories. This method for division makes it easier for the computer to divide data points into large groups based on relationships between many variables. These divisions are easily adjustable as more data comes into the algorithm. When emotional states of animals are set as these divisions through correlation with physiological functions, a support vector machine (SVM) algorithm has the potential to provide a nuanced classification of animal emotional states. This SVM machine learning algorithm can also be used for other aspects of animal welfare, such as predicting sleeping and lying time for cattle and sound analysis [120].

### 5.4. Adoption of Algorithms from Human Biometrics

Because of the social, environment, and economic dimensions, more often the livestock sector embraces innovative technologies developed for human biomedical and space sectors. Recent developments of algorithms in the domain of human biometrics, especially in recognizing human emotions namely, Vortex Optimization algorithm [121]; Black Hole algorithm [122]; Fractal-based algorithm [123]; Adaboost algorithm [124]; Evolutionary computation algorithm [125]; Genetic algorithm [126]; and Deep Convolutional Neural Network Algorithm [127] may find its application in the measurement of animal emotions in the near future.

## 6. Sensor Integration

Currently, a single sensor to address all the needs to cover the emotional spectrum of farm animals is not available. Such a solo sensing platform or a system may not even be possible due to the range of metrics that must be considered. Therefore, at present, the best method to measure emotions and thereby ensure good welfare of animals is a monitoring system that utilizes multiple sensors, each recording different metrics. Sensors working together as a package can provide detailed data on multiple physiological and behavioral parameters, enabling farmers to better maintain the welfare of their stock. To fully comprehend an animal’s emotional state, we probably need to integrate (behavioural and physiological) data from different sensors. Below we propose several combinations of sensor systems that have the potential to optimize animal emotion research in different scenarios. No one of these is a magic bullet—rather, each has its own merits depending on the needs of the farmers and the animals.

### 6.1. Sound Analysis and Facial Expression

Vocalizations and facial expression complement each other well, since animals frequently make distinct facial expressions while vocalizing. Together, these two systems can provide a comprehensive monitoring solution for identifying animals in stress or pain, but also in a positive emotional state. These systems are also both easily implemented through external, mobile camera sensors. The greatest challenge with implementing a system of this nature is establishing the baselines. Highly detailed data on animal sounds and facial expression in reaction to different farm settings and external stimuli must be initially taken before these systems can be effective over the long term. However, once these baselines are established, a system of this nature could prove highly effective for identification of animal emotions and diagnosis of illness and other conditions.

### 6.2. RR, HRV, and Thermal Infrared Imaging

Respiratory rate systems are a good choice for monitoring respiratory and circulatory systems. However, they can be subject to error, so an HRV system is an excellent complement, particularly given the relationship between the heart and the lungs. An additional complement to this system is thermal infrared imaging for recording temperature. These types of data work well together because respiratory and circulatory systems feed into body temperature. There may be connections between these parameters that only become visible once they are monitored simultaneously. Hence along with animal behavioural parameters, fusion of data collected from respiratory rate, heart rate variables, and thermal infrared imaging will provide critical insights about the compound expressions of emotions. By combining the measurement of sounds of animals (vocalization) and facial expressions along with the respiration rate, heart rate variability and thermal infrared imaging compounded emotional expressions of farm animals can be determined.

### 6.3. GPS Tracking Plus Facial Expression and Recognition

GPS and facial expression along with facial recognition serve each other well because GPS can track the location of the animals collectively, while facial expression and recognition can serve to identify individual animals. For this reason, this combination may prove especially useful in medical emergency situations, where facial expression analysis recognizes the issue (e.g., a pain reaction) and GPS works to track the individual animal, allowing the farmer to catch up to the animal and administer the necessary care. This combination of systems is also useful for large flocks of grazing animals, since GPS technology makes it much easier to track animals who have become lost or wandered off.

### 6.4. Facial Expression Using Drones

Stationary cameras are difficult and expensive to implement in exceptionally large farms, since many cameras are required for evaluating emotional states of individual animals in herd. In these types of farm environments, facial expression sensing platforms can be paired well with drone imaging. This further allows facial expression, which generally focuses on individuals, to be optimized for larger scale use [109]. This option is also more affordable, since farmers can purchase a smaller number of cameras than they would need if they were using a stationary system. Unlike stationary cameras, these drone cameras can also be used for security purposes when required.

## 7. Future Perspectives

### Challenges and Opportunities

The main key challenges in enabling the measurement of emotions in farm animals using sensors are practical applicability, costs, feasibility for deployment, quality, and more importantly the validated emotions. Developing sensing methodologies for analyzing the emotions of animals is a delicate art. Among the difficulties faced by researchers in this field is the difficulty in establishing benchmarks. Gathering sufficient data from a wide variety of individuals to establish a “normal” state against which to compare experimental animals can be tremendously time and resource-intensive. Further, it is not just the initial data collection that poses challenges. Algorithms for data analysis and continuous monitoring systems are equally important in processing the emotions of farm animals. However, modern technology can help curb some of the challenges of dealing with such immense pools of data. For example, cloud computing provides an optimal storage solution that is far more efficient than analogue or even disc-based data storage. Cloud-based storage can be integrated with smartphone apps to create a highly secure system that stores data, pulls it from the cloud when required, and analyzes it [128].

There are many challenges that have yet to be fully addressed in assessing the emotions of farm animals. Given the present state of technology, the most effective solution is to customize the emotional analysis and measurement for each farm animal species; however, this is extremely time and resource intensive. These challenges are only worsened for more complex modes of analysis, like facial expression and sound analyses. In order for the systems to be effective, every nuance of the animal’s face or voice must be well-understood in the normal state before analyses can even proceed. And these analyses must be extensive, rigorous, and ongoing to ensure the quality of the overall system.

All of these difficulties presume that the sensor data is of high quality. Increasing reliance on external sensors, as opposed to invasive electrodes, means that sensor output quality is a barrier to their use. Animals cannot be easily instructed to sit still or pose, so these systems are typically working against a backdrop of excess movement. Similar interference occurs in other systems (e.g., ambient noise in sound analysis). There is also a dire need for validated biomarkers to provide evidence of biochemical signatures and their relationship to emotions in livestock animals. This need has the potential to stimulate the deployment of new on-the-spot handheld sensors for measuring these biochemicals, and these sensors are wholly lacking in present commercial products. As the technologies evolve, these systems will ideally be able to measure multiple parameters, such as infrared thermography, to measure temperatures on the face regions, such as snout and eyes, along with biochemical scanners that would measure biomarkers remotely through hair, skin, and nasal or vaginal discharge to provide an indication of the emotional state of the animal.

In evaluating the methods for emotion recognition in humans based on facial and vocal features, Ley et al. [129] explored several modalities and their limitations, benefits, and accuracy, including facial and speech recognition, electromyography, electroencephalography, and electrocardiography. Except for facial and vocal recognition as a modality, none of the other modalities attempted in humans have been explored as a possibility for emotion measurement in animals, mainly due to their complex installation, difficult setup, and lack of validated bio-signals.

With the advent of novel infrared thermal imaging systems and wearable respiration rate sensors, we foresee that it is possible to use respiration along with skin temperature as another factor in determining emotions. The temperature changes surrounding the snouts of pigs, the oral/nasal region of dairy cows, and the eye/beak regions of chickens could possibly serve as a portal for determining the changes in stress levels and other emotions of these farm animals. Similarly, electrodermal activity, heart rate variability measurement through electrocardiography, and electromyography could evolve as new ways of measuring emotions with the advent of non-invasive novel wearable sensors. These new sensors should overcome several challenges, such as the allowing of free movement of animals, overcoming complex installation and movement artifacts, overcoming barriers of background noise in barns, enabling fast signal acquisition to slow occurring bio signals and the ability to distinguish different emotions, and ability to be deployed in a multi-modal setup.

A key challenge in processing the emotions of farm animals in real-time in animal husbandry and barn settings is to identify individual animals. Especially in the case of sound recording, thermal camera, and visual sensors, locating or recognizing individual animals in a herd becomes challenging. This could be overcome by the integration of wearable sensors along with harnessing ‘YOLO’ and machine learning algorithms of facial recognition sensing systems.

The net result of these barriers has been a relative lack of available commercial products. Many of these projects are still in the research phase, meaning the sample set is prohibitively small as far as determining their effectiveness and use. At the present stage of development, comprehensive emotional analysis systems to improve animal welfare are often too expensive for farmers, further limiting the available pool of data.

## 8. Research Directions

**Figure 2:**
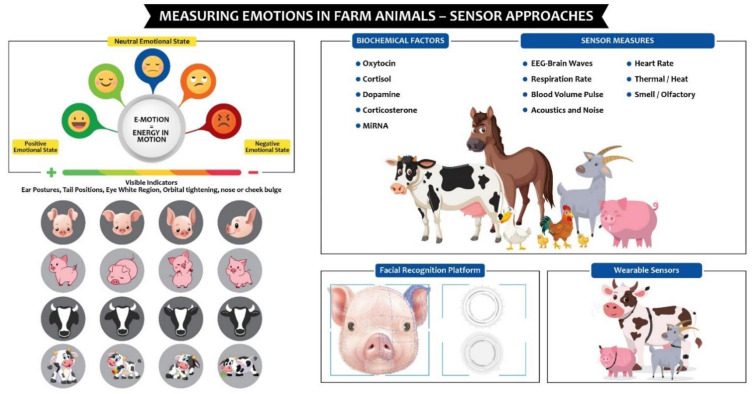
Potential of sensor technologies in measuring farm animal emotions.

Overall, this review demonstrates that current research shows tremendous potential in sensor technology for analyzing the emotions of farm animals (Figure 2). However, that potential has yet to be fully actualized. There are still aspects of this field of study that have not been adequately explored.

First is the potential to develop new and better sensors to simplify the most time-consuming aspect of these systems: baseline data collection. In the future, more sensors can hopefully be streamlined to measure sweat, non-contact vital signals, heart rate variability, respiration rate, temperature, detect pH changes, alert to the presence of virus and bacteria, and measure other metrics not yet considered. The consolidation and packaging of multiple sensors for use by researchers and managers is the next logical step in the development of sensor technology and making these systems affordable for the average user. Moreover, there is a continual need to improve the accuracy of the sensor systems available to us, since incorrect sensor data can result in misdiagnosis of emotion and illness, decreased productivity, or even loss of life.

Strides in the development of technology, specifically in the fields of bioinstrumentation, biosensors, and artificial intelligence will result in numerous new technologies for implementation in humans and animals [19]. This research has been motivated largely by growing concerns about tracking and monitoring the health of farm animals, as well as the current generation of farmer’s growing dependence on technology. Some examples of developing commercial technologies in this space are summarized in Table 4.

While these examples are promising, they demonstrate that at present, commercial sensor products for farm animals are limited. As in any developing technology, only further research and development will result in commercial availability of these systems. More broadly, research into the role of animal emotions and thereby welfare must continue. Historically, welfare has focused more on the absence of negative conditions, but as the connections between positive emotional states and good animal welfare become clearer, it becomes ever more important to develop new and innovative methods to make assessing animal welfare a priority, rather than simply providing adequate conditions.

Questions for future research directions are: Can we infer and predict the emotions of farm animals at multiple dimensions using sensor-based data? What level of sensor fusion is required in differentiating the individual dimensions of the farm animal activity and the behavior based on emotion measurements? Which combination of sensors as wearables and sensors for measuring environmental parameters can automatically predict the animal behavior and emotional states in real farm scenarios? What are the correlating factors that enable the multidimensional animal behavior and animal emotions for translating into physiological functions? Can emotions of farm animals be used as assets in the creation of digitalized duplicates of animals (Digital Twins of Animals) using sensors?

Research into animal emotion is not distinct from the development of technology, but rather is an essential complement to it. This research provides the theoretical background for the implementation of technological systems. While there is currently no single infallible system for monitoring farm animals’ emotions, the coming years will likely see an explosion of these technologies. But for now, much remains to be explored.

## 9. Conclusions

Emotion detection is an important aspect of animal welfare, and in the absence of verbal communication, we must continue to rely on the measurement of physiological and behavioral parameters to attempt to illuminate the feeling component of emotion. Sensors represent the present and the future in technological solutions for management of livestock. Sensing platforms offers the possibilities of automated and continuously monitoring the behavior of livestock through emotion measurement techniques. As we have reviewed in this paper, there are limited applications of sensors available for the monitoring of emotions of farm animals. Despite these current limitations, new sensing technologies will likely have a massive role to play in the future of digitalization of agriculture, including animal emotions. With the advent of industry 4.0 and agriculture 5.0, artificial intelligence and sensor-based facial recognition platforms will become an everyday tool to enable farmers in predicting behavior of the livestock through precise measurement of emotions.

## Figures and Tables

**Table 1:** Summary of emotions expressed by farm animals and sensing parameters related to recognize each emotion.

Farm Animal Species	Indicators Inferring Emotions and the Emotions/Affective States	References
Sheep	Horizontal ear posture—Neutral state Ears backward—Fear Ears up—Anger Asymmetric ears—Surprise	[15]
Sheep	Ear flat—Pain not present Ear flipped—Pain fully present/Negative state Shallow U-shaped nose—Pain not present/Neutral or positive state Extended V shaped nose—Pain fully present/Negative state Eye fully open, Pain fully present/Negative state Eye partly closed, Pain not present, Neutral or positive state	[24,25]
Lamb	Cheek flattening—Less bulging of nose and cheek area—Pain Ear Posture—Ears tense and point backwards or downwards (no visible inner ear)—Pain Ears relaxed and horizontal or inner ear visible—Not in pain or neutral state Flat and tight lip like horizontal line—‘Smile’ emotion and not in pain Tight nose with decreased nostril size—Pain V shape nose—in pain—U shape nose—Not in pain Squeezing or closing of eye (Orbital tightening)—In Pain	[26]
Goat	Ears lowered and turn down—Positive emotions Ear tips pointing backwards, and auricles turned down—Negative emotions	[27]
Horse	Lower oxytocin level—Neutral and positive emotional states Rise in cortisol levels and rise in heart rate parameters—Stress	[28]
Horse	More eye white region—Negative emotion experience Decrease in eye wrinkle expression—Positive emotion condition Increase in eye wrinkle expression—Increase in negative emotion	[29]
Horse	Increase in spontaneous blink rate of eye—Stress Increase in dopamine levels—Positive emotion due to reward Increase in salivary cortisol and change in heart rate variability—Stress	[30]
Horse	Increase in heart rate, eye white increase, nostril dilator, upper eyelid raiser, inner brow raiser, tongue show with increase in ear flicker and blink frequency—All related to increase in stress	[31]
Cow	Upright ear posture longer—Excitement Forward facing ear posture—Frustration	[32]
Cow	Half-closed eyes and ears backwards or hung-down—Relaxed State Eye white clearly visible and ears directed forward—Excited State	[33]
Cow	Decrease in nasal temperature and change in peripheral temperature—Positive experience or increase in arousal	[34]
Cow	Cow vocalizations—Open mouth calls & a greater number of vocal units per sequence—alert and stress escalation Close mouth calls—Positive emotional state	[35]
Cow	Visible eye white and maximum eye temperature—Stress	[36]
Dairy Calves	Lower heart rate—positive emotion Higher heart rate—negative emotion Increase in salivary cortisol—Both positive and negative emotion Higher secretory immunoglobulin A (SIgA), Serum IL-2 and IL-3 levels—Positive emotional states Higher serum of tumor necrosis factor alpha (TNFα)—Negative emotion	[37]
Hens	Increase in cortisol in serum—Negative emotions and stress Increase in corticosterone levels in feathers—Positive emotions	[38]
Hens	Increase in corticosterone levels in feathers—Positive excited states	[39]
Chickens	Tachycardia and bodily fever—Fear Increased locomotion and pacing behaviour—Anxiety or Negative emotion Lower corticosterone—Positive emotion	[40]
Chickens	Repetitive, high energy calls (sounds)—Distress or negative emotions	[41]
Pigs	High frequency ear movement—Stress or negative emotion High duration lateral tail movement—Positive emotions or play behavior	[42]
Pigs	Tail raised and forming a loop—Positive emotion Ears forward—Alert and neutral emotion Ears backward—Negative emotion Hanging ears flipping in the direction of eyes—Normal state (Neutral emotion Standing upright ears—Normal neutral state	[43,44]
Pigs	Smaller snout ration and ears forward oriented—Aggression or negative emotion state Ears backward and less open eyes—Retreat from aggression or transition to neutral state	[45]
Pigs	Tail hang loose—Negative or neutral emotion state	[46]
Pigs	Curled up tails and ears directed forward—Positive emotion state Tucked under tails—Negative emotion	[47]

**Table 2:** Pros and cons of different sensor systems related to emotions measurement.

System	Pros	Cons
Global Positioning System	Long-lasting system, noninvasive	Expensive at startup, battery life, issues with accuracy, noise
Thermal Infrared Imaging	Accurate indicator of temperature, noninvasive	Subject to interference from external heat sources
Electrocardiograph	Likely reliable indicator of positive affect through heart rate measurement	Deployability issues due to motion artefacts; Not practical for real-time or on-site monitoring
Electroencephalography	Accurate measure of brain activity irrespective of subject movement	Dissociation between EEG states and emotional valences; Real-time non-invasive sensors are not yet available
Electromyogram	Useful for many diagnostics	Subject to interference; Only measures surface muscles
Respirometer	Especially useful for diagnostics and for animals with distinct breath patterns	Difficult to implement and influenced by many factors including motion
Olfactory and chemical sensors	Strongly linked to emotion	Do not use data from the animal directly; Indirect measurement as validated benchmarks is unavailable

**Table 3:** Key features of machine learning algorithm systems.

System	Features
Neural Networks	Employs many distinct algorithms together; Can mimic the brain of the target species
Fuzzy Logic	Compares continuous inputs to known baselines
Support Vector Machine Algorithms	Useful in classification; good for establishing broad relationships between many variables

**Table 4:** Commercial technologies for animal monitoring.

Company	Project
Iris Data Science, New Zealand	Prototyped sheep reidentification system designed to be affordable for farmers
Moofarm, India	Cattle reidentification system
SmartAHC, Singapore	Livestock management technologies for record-keeping, facial recognition, health management, and quality assurance
Yingzi Technology, China	Facial recognition system for pigs used for tracking, preventing meat fraud, and working towards productivity

## Data Availability

Data sharing is not applicable to this article.
